# Epidemiology of COVID-19 infections on routine polymerase chain reaction (PCR) and serology testing in Coastal Kenya

**DOI:** 10.12688/wellcomeopenres.17661.1

**Published:** 2022-02-23

**Authors:** James Nyagwange, Leonard Ndwiga, Kelvin Muteru, Kevin Wamae, James Tuju, COVID testing team, Bernadette Kutima, John Gitonga, Henry Karanja, Daisy Mugo, Kadondi Kasera, Patrick Amoth, Nickson Murunga, Lawrence Babu, Edward Otieno, George Githinji, D.J. Nokes, Benjamin Tsofa, Benedict Orindi, Edwine Barasa, George Warimwe, Charles N. Agoti, Philip Bejon, Lynette Isabella Ochola-Oyier

**Affiliations:** 1 Kenya Medical Research Institute, Kilifi, Kenya; 2 Ministry of Health, Nairobi, Kenya

**Keywords:** SARS-CoV-2, COVID-19, epidemiology, serology, RT-PCR, ELISA, clinical characteristics

## Abstract

**Background:** There are limited studies in Africa describing the epidemiology, clinical characteristics and serostatus of individuals tested for severe acute respiratory syndrome coronavirus 2 (SARS-CoV-2) infection. We tested routine samples from the Coastal part of Kenya between 17 ^th^ March 2020 and 30 ^th^ June 2021.

**Methods:** SARS-CoV-2 infections identified using reverse transcription polymerase chain reaction (RT-PCR) and clinical surveillance data at the point of sample collection were used to classify as either symptomatic or asymptomatic. IgG antibodies were measured in sera samples, using a well validated in-house enzyme-linked immunosorbent assay (ELISA).

**Results: **Mombasa accounted for 56.2% of all the 99,694 naso-pharyngeal/oro-pharyngeal swabs tested, and males constituted the majority tested (73.4%). A total of 7737 (7.7%) individuals were SARS-CoV-2 positive by RT-PCR. The majority (i.e., 92.4%) of the RT-PCR positive individuals were asymptomatic. Testing was dominated by mass screening and travellers, and even at health facility level 91.6% of tests were from individuals without symptoms. Out of the 97,124 tests from asymptomatic individuals 7,149 (7%) were positive and of the 2,568 symptomatic individuals 588 (23%) were positive. In total, 2458 serum samples were submitted with paired naso-pharyngeal/oro-pharyngeal samples and 45% of the RT-PCR positive samples and 20% of the RT-PCR negative samples were paired with positive serum samples. Symptomatic individuals had significantly higher antibody levels than asymptomatic individuals and become RT-PCR negative on repeat testing earlier than asymptomatic individuals.

**Conclusions:** In conclusion, the majority of SARS-CoV-2 infections identified by routine testing in Coastal Kenya were asymptomatic. This reflects the testing practice of health services in Kenya, but also implies that asymptomatic infection is very common in the population. Symptomatic infection may be less common, or it may be that individuals do not present for testing when they have symptoms.

## Introduction

Severe acute respiratory syndrome coronavirus 2 (SARS-CoV-2) infection began in Wuhan, China in December 2019 and rapidly spread around the world causing a disease called coronavirus disease 2019 (COVID-19) ( Cucinotta & Vanelli, 2020). Patients with COVID-19 present with a wide range of symptoms such as fever, cough, shortness of breath, headache, body-aches, fatigue, loss of taste and smell, conjunctivitis, and diarrhea ( Guan *et al*., 2020). The World Health Organization (WHO) declared the disease a pandemic on March 11 ^th^ 2020 and two days later 13 ^th^ March 2020, the first case of COVID-19 was detected in Nairobi, Kenya ( MoH, 2020). In the Kenyan Coast, the first case was detected in Mombasa county on 21 ^st^ March 2020. By 30 ^th^ June 2021, the total number of confirmed cases in Kenya was 95,843, with 1,655 deaths ( WHO, 2021). In 2020 and up to June 2021, Kenya experienced three waves; the first wave was in July - August 2020, the second wave observed shortly after from November - December 2020 attributable to the epidemic spreading to new socio-economic groupings, and a third wave from April-May 2021 attributable to variants of concern ( Brand *et al*., 2021).

The KEMRI-Wellcome Trust Research Programme (KWTRP) is a government designated SARS-CoV-2 testing laboratory for the Coastal region of Kenya that includes six counties namely, Mombasa, Kilifi, Lamu, Taita-Taveta, Tana River and Kwale. The KWTRP laboratory received nasal-oropharyngeal (NP/OP) samples from several hospitals and clinics for acute testing and screening of contacts and occasionally, serum and plasma samples for serology.

There is no published detailed description of the epidemiology and clinical characteristics of SARS-CoV-2 infections in Kenya. Here we report the epidemiology and symptoms for individuals in Coastal Kenya who were tested at (KWTRP), in Kilifi. In a sub-set of the samples, based on sera availability, we also describe the temporal seroprevalence of anti-SARS-CoV-2 IgG and examine the impact of age, sex, viral load and infection status on the time to clear infections.

## Methods

### Study population and data collection

We conducted a retrospective analysis of surveillance data obtained between 17 ^th^ March 2020 and 30 ^th^ June 2021 from the Coastal part of Kenya, including Mombasa, Taita Taveta, Kilifi, Kwale, Lamu and Tana River. Trained public health rapid response team members and healthcare personnel investigated suspected COVID-19 cases based on contact tracing and history of travel to endemic regions, completed a detailed case investigation form (CIF) and collected a nasopharyngeal and oropharyngeal swab in viral transport media. Collected specimens were triple-packed and transported in cold chain (4°C) to KWTRP where the RT-PCR testing was performed. The data from all the CIFs were entered into Research Electronic Data Capture ( REDCap version 10.5.1, RRID:SCR_003445), a web application at KWTRP. COVID-19 test results were relayed daily to the Ministry of Health following their guidelines. The CIF can be found as *Extended data* ( Nyagwange *et al*., 2022b).

### Data cleaning

The COVID-19 surveillance dataset from 17 ^th^ March 2020 up to 30 ^th^ June 2021 covering the three epidemic waves was systematically curated to ensure uniform entries on the sex categorical variable, all participants had their ages in years and proper spelling of the county of residence variable. For each participant, the timepoint used for analysis was the first positive test. An asymptomatic episode was defined as being positive (by RT-PCR) for COVID-19 at the time of sampling, without any of the 14 symptoms: fever (temperature ≤ 37.5°C), cough, general weakness, history of fever, headache, sore throat, shortness of breath, runny nose, chest pain, nausea, muscular pain, diarrhea, irritation, joint pain and abdominal pain. A symptomatic episode was defined as being positive for COVID-19 at time of sampling, with the above-mentioned symptoms.

### RT-PCR testing

SARS-CoV-2 RNA was extracted from NP/OP samples using available commercial kits: QIAamp Viral RNA Mini Kit (Qiagen, Catalog Number: 52906), DAAN kit (DAAN Gene Co., Ltd of Sun Yat-sen University, Catalog Number: DA-0591) and SpinX (TCG Pharma Inc., Catalog Number: PPT-TN04) according to the manufacturer’s protocol. To identify SARS-CoV-2 infections, RT-PCR amplifications were done using ABI real-time system, model 7500 (Applied Biosystems, Warrington, United Kingdom) with a number of kits as described before ( Said *et al*., 2020). The primer-probe sequences and RT-PCR cycling conditions have been described elsewhere and the primers and probes generally target RNA dependent RNA polymerase (RdRp), envelope (E), (ORF)1ab and nucleocapsid (N) regions ( Said *et al*., 2020). Two negative and positive controls were included in each run for quality control.

### Spike antigen production and ELISA assay

We have recently described the production and purification of full-length SARS-CoV-2 spike protein in mammalian expression system ( Uyoga *et al*., 2021). The IgG antibodies were measured using a previously described ELISA assay developed and validated at KWTRP, Kilifi, Kenya with sensitivity of 92.7% (95% CI 87.9–96.1%) and specificity of 99.0% (95% CI 98.1–99.5%) ( Uyoga *et al*., 2021). The assay has also been validated in a WHO sponsored multi-laboratory study of SARS-CoV-2 antibody assays and performed as well as the other international laboratories ( Adetifa *et al*., 2021) and as well as WHO endorsed commercial ELISA ( Nyagwange *et al*., 2022a).The sample results were expressed as the optical density (OD) ratio, which is a ratio of test OD to the OD of the plate negative control; OD ratio greater than two was considered seropositive for SARS-CoV-2 IgG ( Uyoga *et al*., 2021).

### Statistical analysis

IgG antibody responses were compared between asymptomatic and symptomatic groups using the Wilcoxon test. Participants were stratified into two groups based on age (≤20 and >20 years) as there was a skew towards participants of younger age groups pre-COVID-19. Temporal changes in Ct values and IgG responses were examined using the Wilcoxon and Kruskal-Wallis tests. Differences in Ct value by clinical status (asymptomatic vs. symptomatic) and by age group were compared using the Wilcoxon and Kruskal-Wallis tests. Survival analysis was used to compare *(i)* the time to the first negative COVID-19 test between asymptomatic and symptomatic patients, *(ii)* the time taken to the first negative COVID-19 test in asymptomatic individuals, stratified by age (>0–20, >20–50 and >50 age groups). Patients who did not complete the follow up, were censored. All statistical analyses were conducted in R v4.0.2 ( R Core Team, 2013, RRID:SCR_001905), all plots were generated using the packages ggplot2 v3.3.2 ( Wickham, 2016) and ggpubr v0.4.0. Survival analysis was performed using the survival package v3.2-7 ( Therneau & Grambsch, 2000; Therneau, 2020) and Kaplan-Meier plots were generated using the survminer package v.0.4.8 ( Kassambara *et al*., 2018). The survival curves were compared using log-rank test. A linear regression model was used to determine whether age, sex, infection status i.e., asymptomatic or symptomatic and viral load cause a change in the OD ratio. The analysis code can be found as *Extended data* ( Nyagwange *et al*., 2022b).

### Ethical approval

This study was approved by the Kenya Medical Research Unit- Scientific Ethic Review Committee (KEMRI-SERU) under protocol number SERU4081: Integrated studies of the natural history of Sars-Cov-2 infections in Kenya.

## Results

### General characteristics of the study population

A total of 99,694 individuals were tested for SARS-CoV-2 from the six Coastal counties of Kenya between 17 ^th^ March and 30 ^th^ June 2021. Majority of the samples were from Mombasa (49.3%), followed by Kilifi (22.1%), Taita-Taveta (17.3%), Kwale (7.1%), Lamu (3.1%) and Tana River (0.9%) ( Table 1). The majority of tested individuals were male (73%), with the most common age bracket tested being between 30 and 40, 30.4% ( Table 1). The median age was 35 years, with the youngest and oldest participants being 7 months and 105 years old, respectively.

**Table 1.** The baseline characteristics of the study population across the Coastal counties.

	MOMBASA (N=49173)	KILIFI (N=22080)	TAITA TAVETA (N=17267)	KWALE (N=7058)	LAMU (N=3127)	TANA RIVER (N=901)	(MISSING) (N=88)	TOTAL (N=99694)
**SEX**								
** (MISSING)**	2377 (4.8%)	875 (4.0%)	431 (2.5%)	734 (10.4%)	124 (4.0%)	35 (3.9%)	83 (94.3%)	**4659 (4.7%)**
** FEMALE**	9252 (18.8%)	7325 (33.2%)	3411 (19.8%)	595 (8.4%)	865 (27.7%)	372 (41.3%)	2 (2.3%)	**21822 (21.9%)**
** MALE**	37544 (76.4%)	13880 (62.9%)	13425 (77.7%)	5729 (81.2%)	2138 (68.4%)	494 (54.8%)	3 (3.4%)	**73213 (73.4%)**
**AGE (YEARS)**								
** (MISSING)**	884 (1.8%)	944 (4.3%)	264 (1.5%)	174 (2.5%)	64 (2.0%)	8 (0.9%)	84 (95.5%)	**2422 (2.4%)**
** >0–20**	4119 (8.4%)	2668 (12.1%)	1051 (6.1%)	412 (5.8%)	242 (7.7%)	201 (22.3%)	0 (0.0%)	**8693 (8.7%)**
** >20–30**	11736 (23.9%)	5708 (25.9%)	5045 (29.2%)	1986 (28.1%)	1148 (36.7%)	240 (26.6%)	1 (1.1%)	**25864 (25.9%)**
** >30–40**	14559 (29.6%)	5926 (26.8%)	5654 (32.7%)	2295 (32.5%)	845 (27.0%)	214 (23.8%)	0 (0.0%)	**29493 (29.6%)**
** >40–50**	11351 (23.1%)	4104 (18.6%)	3299 (19.1%)	1484 (21.0%)	501 (16.0%)	103 (11.4%)	3 (3.4%)	**20845 (20.9%)**
** >50**	6524 (13.3%)	2730 (12.4%)	1954 (11.3%)	707 (10.0%)	327 (10.5%)	135 (15.0%)	0 (0.0%)	**12377 (12.4%)**

The most common source of the tests was from mass testing exercises (28.3%), followed by truck drivers (27.6%), and healthcare facilities (11.4%). However, 30.2% of tests had missing information on the source ( Table 2). 

**Table 2.** Baseline Characteristics of individuals based on their reason for testing.

	(MISSING) (N=30095)	HEALTH FACILITY ^A^ (N=11374)	MASS TESTING ^B^ (N=28237)	STUDY_ RELATED ^C^ (N=1215)	AIR TRAVELLER (N=1293)	TRUCK DRIVER ^D^ (N=27480)	TOTAL (N=99694)
**SEX**							
** (MISSING)**	2390 (7.9%)	466 (4.1%)	937 (3.3%)	76 (6.3%)	30 (2.3%)	760 (2.8%)	4659 (4.7%)
** FEMALE**	7424 (24.7%)	4741 (41.7%)	8940 (31.7%)	241 (19.8%)	258 (20.0%)	218 (0.8%)	21822 (21.9%)
** MALE**	20281 (67.4%)	6167 (54.2%)	18360 (65.0%)	898 (73.9%)	1005 (77.7%)	26502 (96.4%)	73213 (73.4%)
**AGE (YEARS)**							
** (MISSING)**	1222 (4.1%)	483 (4.2%)	422 (1.5%)	85 (7.0%)	32 (2.5%)	178 (0.6%)	2422 (2.4%)
** >0–20**	3182 (10.6%)	1448 (12.7%)	3615 (12.8%)	30 (2.5%)	88 (6.8%)	330 (1.2%)	8693 (8.7%)
** >20–30**	9392 (31.2%)	3091 (27.2%)	7826 (27.7%)	508 (41.8%)	370 (28.6%)	4677 (17.0%)	25864 (25.9%)
** >30–40**	8305 (27.6%)	2952 (26.0%)	7476 (26.5%)	339 (27.9%)	403 (31.2%)	10018 (36.5%)	29493 (29.6%)
** >40–50**	4809 (16.0%)	1759 (15.5%)	5402 (19.1%)	164 (13.5%)	215 (16.6%)	8496 (30.9%)	20845 (20.9%)
** >50**	3185 (10.6%)	1641 (14.4%)	3496 (12.4%)	89 (7.3%)	185 (14.3%)	3781 (13.8%)	12377 (12.4%)
**CLINICAL STATUS**							
** MISSING**	0 (0.0%)	1 (0.0%)	0 (0.0%)	1 (0.1%)	0 (0.0%)	0 (0.0%)	2 (0.0%)
**ASYMPTOMATIC**	29304 (97.4%)	10421 (91.6%)	27537 (97.5%)	1198 (98.6%)	1278 (98.8%)	27386 (99.7%)	97124 (97.4%)
**SYMPTOMATIC**	791 (2.6%)	952 (8.4%)	700 (2.5%)	16 (1.3%)	15 (1.2%)	94 (0.3%)	2568 (2.6%)

^A^ Individuals tested in a health facility, ^B^This covers individuals tested through contact tracing, quarantine and surveillance. ^C^ This group represents samples collected for three studies: Chadox, ImmunoCov and IP. ^D^represents truck drivers, 112/27480 were tested in a health facility.

**Figure 1.**
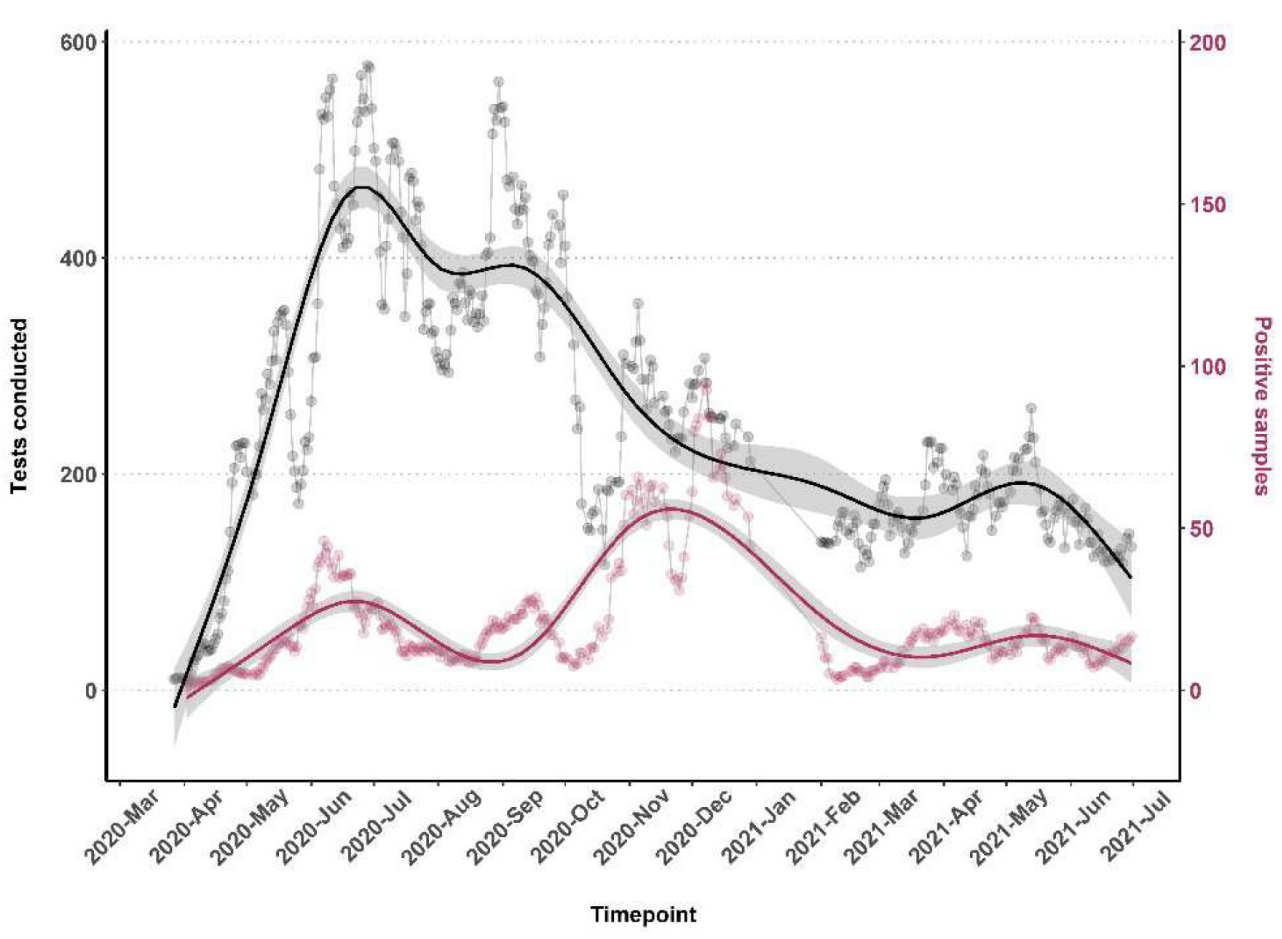
The number of tests done along with the positivity rate from 17th March 2020 - 30th June 2021. The number of tests and positivity rate was calculated using the ‘rolling average method’. This was done by taking the average value of 7 days to determine trends that would otherwise be difficult to detect.

The bulk of the testing was conducted between May and December 2020 ( Figure 1). Cumulatively, testing increased steadily from March and peaked in June 2020 before declining and rising slightly in August-September 2020 and November 2020 and thereafter in May 2021 ( Figure 1).

### Characteristics of the SARS-CoV-2 positive population

**Figure 2.**
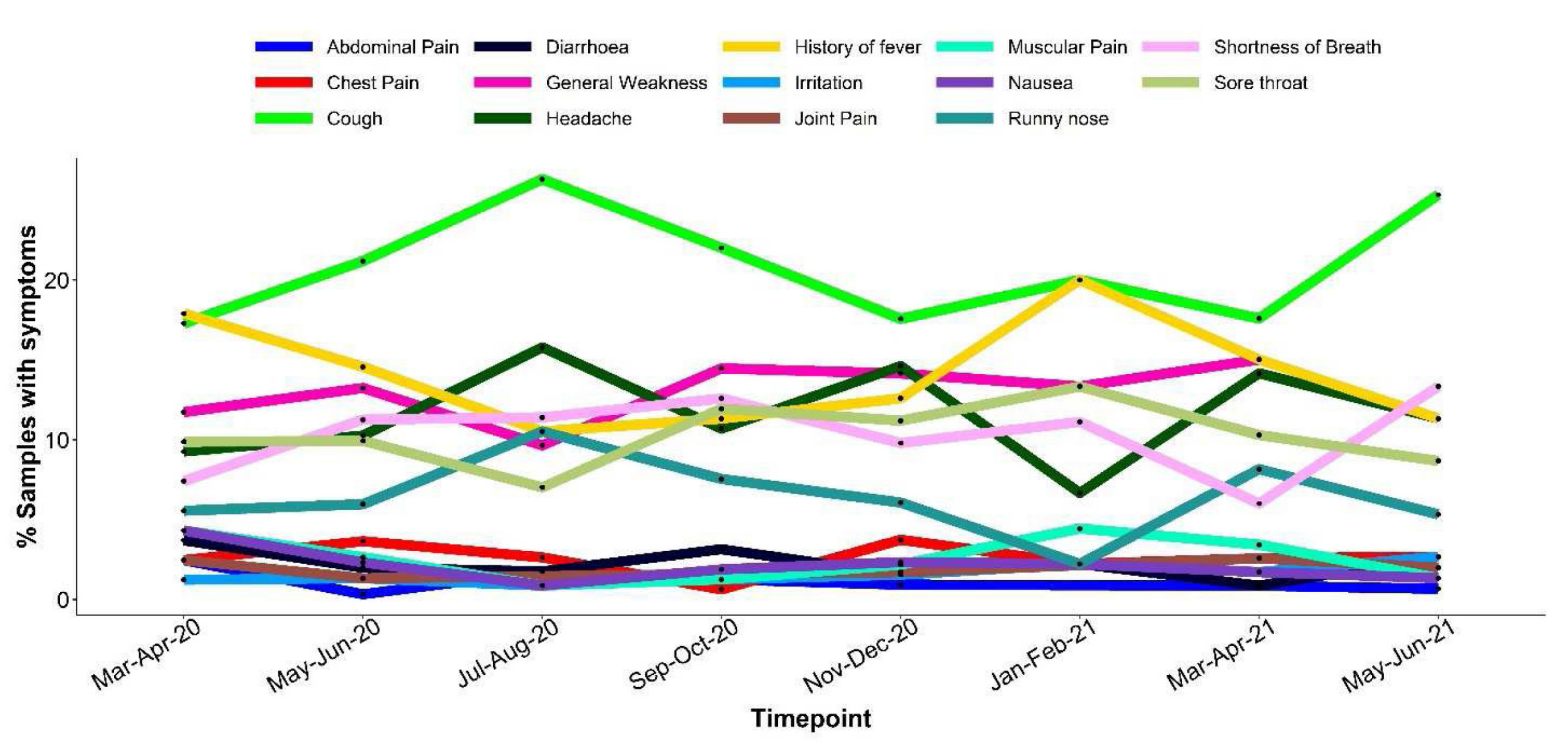
Graph showing bi-monthly distribution of the 14 symptoms reported between March 2020 and June 2021.

Of the 99,694 samples tested 7,737 (7.7%) were SARS-CoV-2 positive by RT-PCR. A closer analysis of all individuals who tested positive for SARS-CoV-2, revealed majority (64%) were males ( Table 3). Within the health facility, 91.6% of tests were from asymptomatic individuals (presumably contacts of cases), and the other sources of tests (including mass screening and travelers’ tests) were mostly of asymptomatic individuals. In total 97.4% of all tests were from asymptomatic individuals. Out of the 97,124 tests from asymptomatic individuals 7,118 (7%) were positive and of the 2,568 symptomatic individuals 619 (24%) were positive. Among the symptomatic cases, the most reported symptoms across the months were cough (19.6%), general weakness (13.4%), history of fever (13.3%), headache (12.8%), sore throat (10.4%), shortness of breath (10.2%) and runny nose (6.5%) ( Figure 2). Other less common symptoms were chest pain (3.1%), nausea (2.4%), muscular pain (2.3%), diarrhea (2.1%), irritation (1.4%), joint pain (1.4%) and abdominal pain (1.1%) ( Figure 2). Symptoms were more common among older participants, Table 3.

**Table 3.** The distribution of asymptomatic and symptomatic cases for each sex and age group.

	ASYMPTOMATIC (N=7149)	SYMPTOMATIC (N=588)	TOTAL (N=7737)	P VALUE
**SEX**				**< 0.01**
** (MISSING)**	314 (4%)	10 (2%)	324 (4%)	
** FEMALE**	2192 (31%)	263 (45%)	2455 (32%)	
** MALE**	4643 (65%)	315 (54%)	4958 (64%)	
**AGE CATEGORY (YEARS)**				**< 0.01**
** MISSING**	230	13	243	
** >0–20**	883 (13%)	36 (6%)	919 (12%)	
** >20–30**	1924 (28%)	121 (21%)	2045 (27%)	
** >30–40**	1910 (28%)	131 (23%)	2041 (27%)	
** >40–50**	1236 (18%)	117 (20%)	1353 (18%)	
** >50**	966 (14%)	170 (30%)	1136 (15%)	

**Figure 3.**
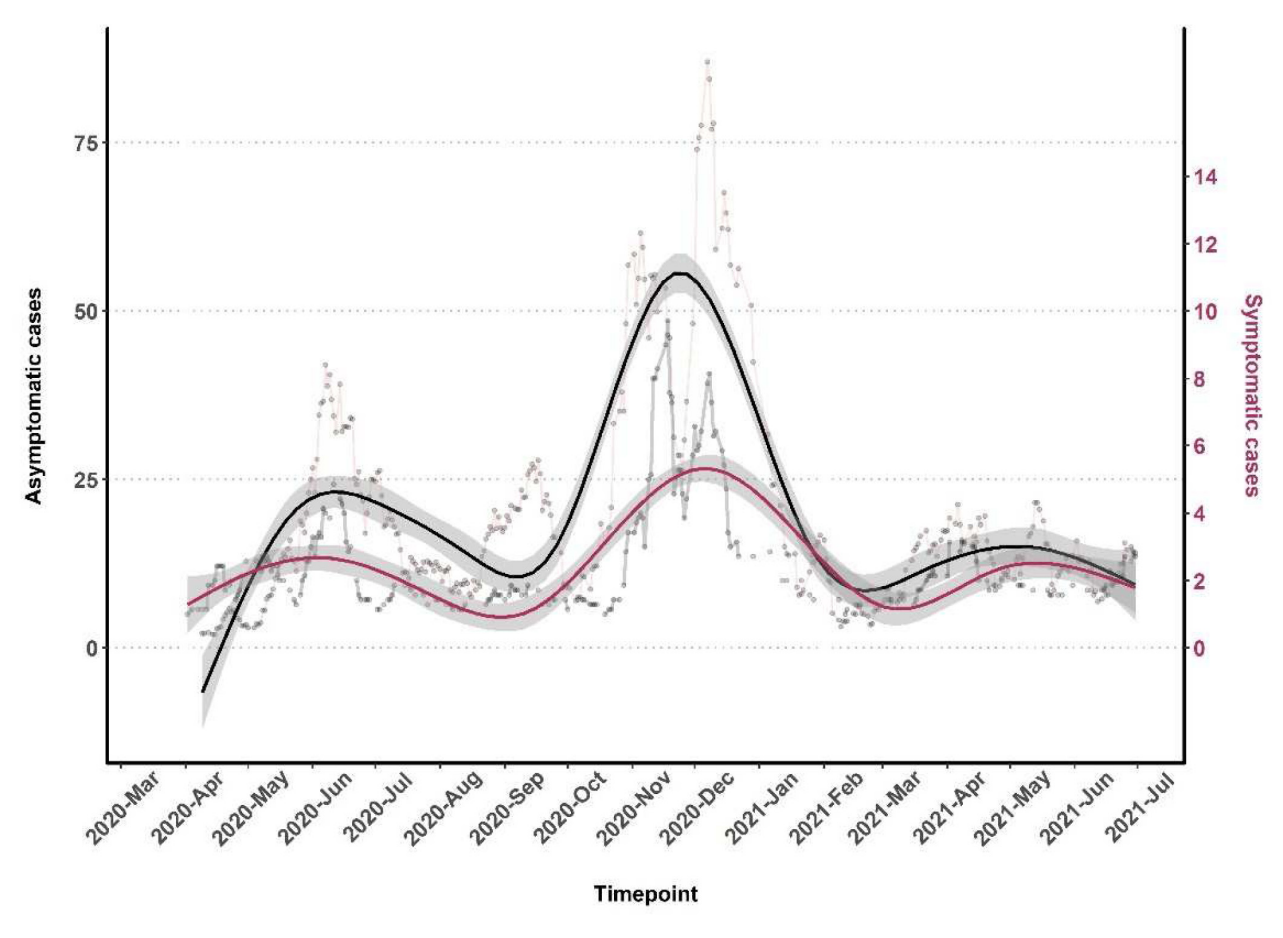
The monthly distribution of Asymptomatic (primary y-axis) and Symptomatic (secondary y-axis) infections in severe acute respiratory syndrome coronavirus 2 (SARS-CoV-2) positive cases.

Throughout the testing period and the three waves of increasing positivity, asymptomatic cases were predominantly observed ( Figure 1 and Figure 3). There was a slight increase in the proportion of symptomatic cases in wave three, 8.2% (986/1051) in comparison to waves one, 7.4% (218/2959), and two, 7.6% (284/3727). There was no trend over time for the specific type of symptom reported ( Figure 3).

**Figure 4.**
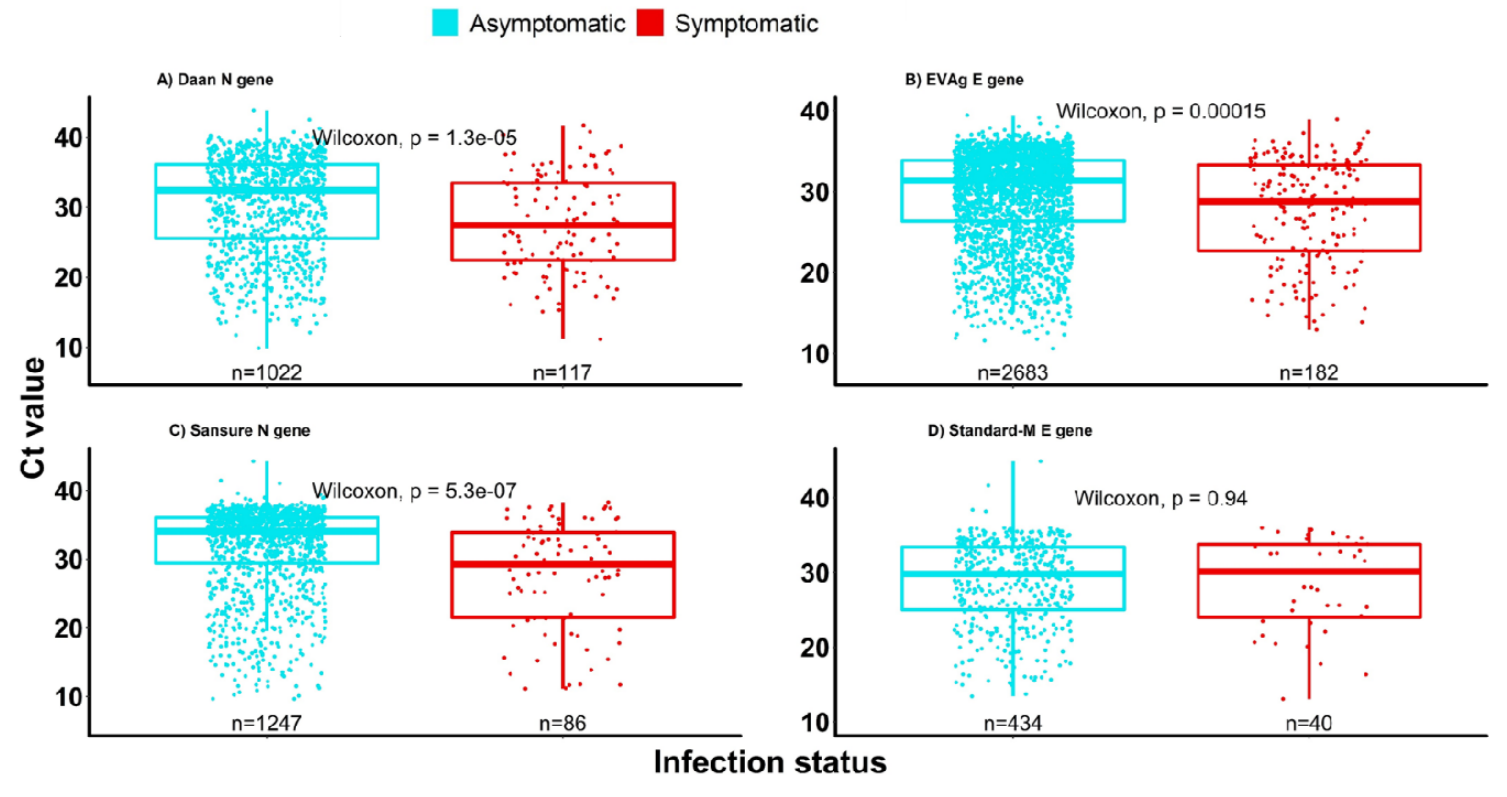
The virological characteristics in asymptomatic and symptomatic severe acute respiratory syndrome coronavirus 2 (SARS-CoV-2) positive cases. The figure shows Ct value data for 7594 of the 7737samples.

**Figure 5.**
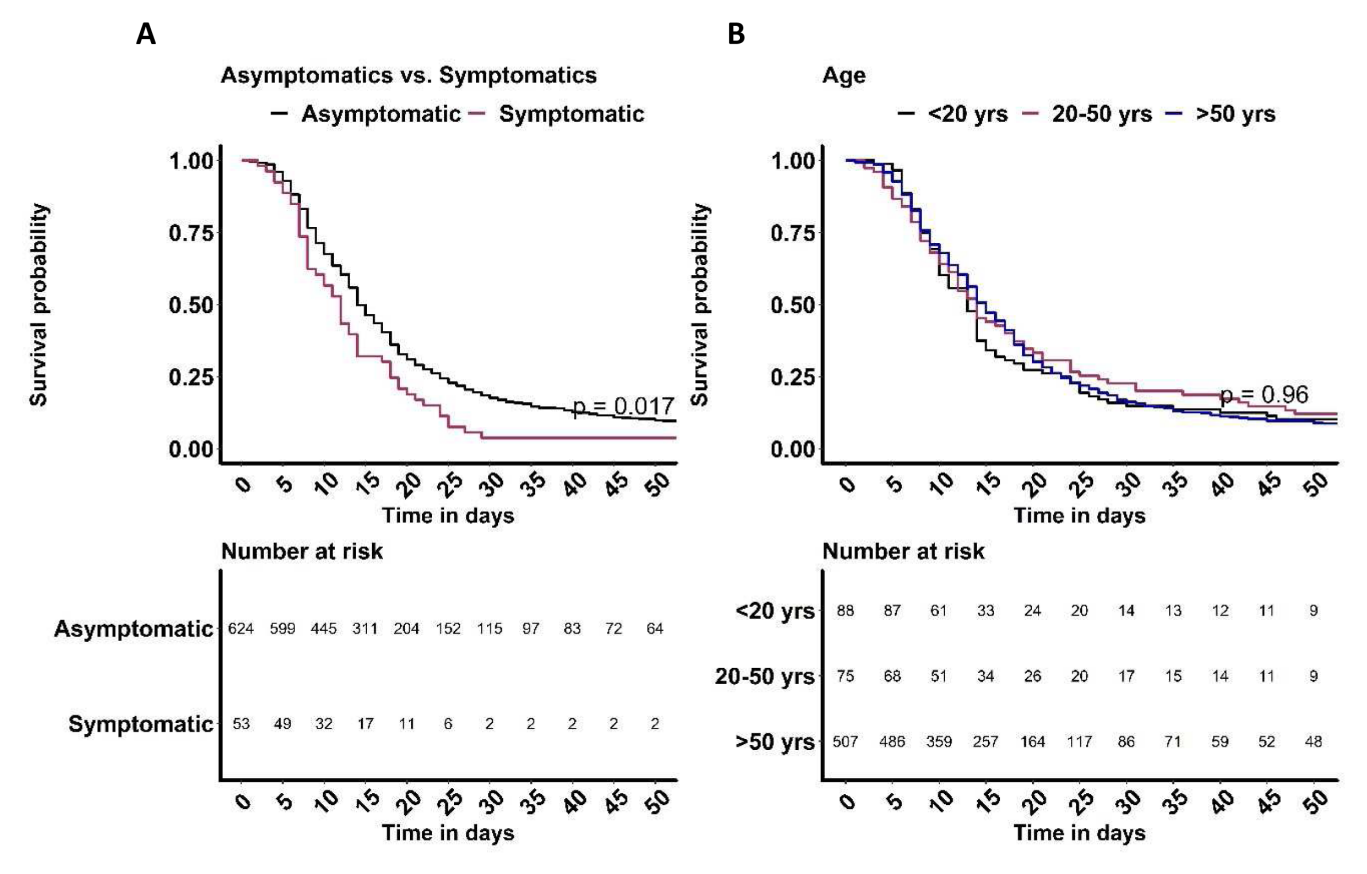
Kaplan Meier plots of time to clearing severe acute respiratory syndrome coronavirus 2 (SARS-CoV-2) infection by ( **A**) clinical status, asymptomatic and symptomatic infections and ( **B**) age (<20 years, 20–50 years and >50 years).

On average, symptomatic individuals had significantly higher viral loads (low Ct values) compared to asymptomatic individuals, irrespective of the RT-PCR assay kit and gene tested ( Figure 4). Based on longitudinal data obtained from individuals with follow up samples, symptomatic individuals cleared the infection faster than the asymptomatic individuals (p=0.017, Figure 5A). However, there was no difference in time taken to clear SARS-CoV-2 infection by age ( Figure 5B).

### Serological analysis

**Figure 6.**
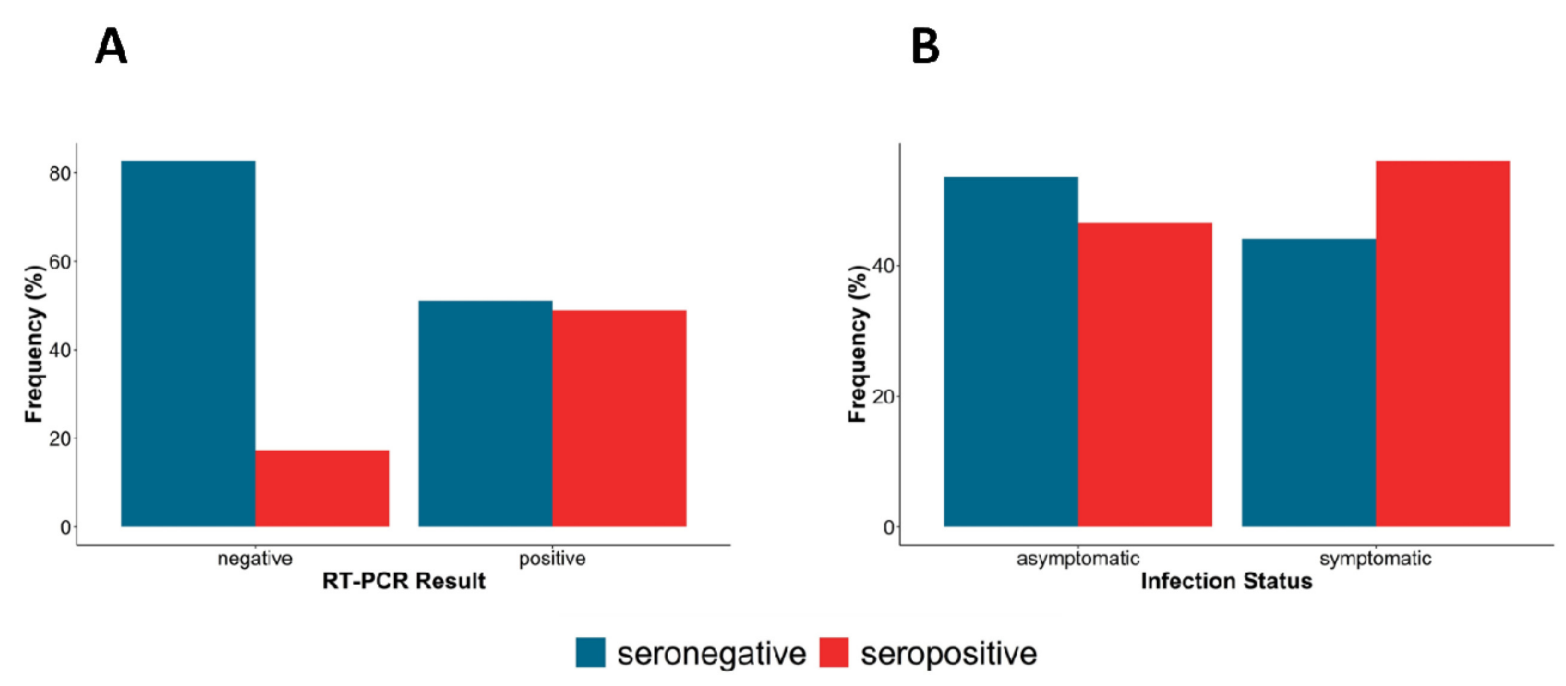
The proportion of IgG seropositivity in ( **A**) *Nasal-oropharyngeal (NP/OP) swab, reverse transcription polymerase chain reaction (RT-PCR)* positives and negatives ( **B**) Asymptomatic and symptomatic infections.

Serum samples were only obtained from March to November 2020 that were used to determine the prevalence of anti‐SARS‐CoV‐2 spike IgG. The overall prevalence of anti‐SARS‐CoV‐2 spike‐IgG was 20.3% (494/2437) and by sampling location were as follows; Kilifi 36.1% (66/183), Lamu 20.8%, (108/518), Mombasa 19.5% (314/1609), Kwale 4.1% (5/123), Taita Taveta 25% (1/4). Seropositivity peaked in August and November 2020, though in the latter month the sample size was small ( Table 4). When stratified by RT-PCR results, a large proportion of RT-PCR positive samples were seropositive (45%) compared to RT-PCR negative samples (20%) and a larger proportion of symptomatic individuals were also seropositive (60%) compared to asymptomatic individuals (45%) ( Figure 6).

**Table 4.** The baseline characteristics of samples used for serology.

	Mar 2020 (N=12)	Apr 2020 (N=434)	May 2020 (N=91)	Jun 2020 (N=1234)	Jul 2020 (N=325)	Aug 2020 (N=117)	Sep 2020 (N=106)	Oct 2020 (N=103)	Nov 2020 (N=15)	Total (N=2437)
**Sex**										
Female	4 (33.3%)	215 (49.5%)	48 (52.7%)	285 (23.1%)	95 (29.2%)	33 (28.2%)	50 (47.2%)	6 (5.8%)	6 (40.0%)	**742 (30.4%)**
Male	8 (66.7%)	200 (46.1%)	41 (45.1%)	929 (75.3%)	215 (66.2%)	83 (70.9%)	56 (52.8%)	92 (89.3%)	3 (20.0%)	**1627 (66.8%)**
(Missing)	0 (0.0%)	19 (4.4%)	2 (2.2%)	20 (1.6%)	15 (4.6%)	1 (0.9%)	0 (0.0%)	5 (4.9%)	6 (40.0%)	**68 (2.8%)**
**Age (years)**										
>0–20	1 (8.3%)	29 (6.7%)	4 (4.4%)	61 (4.9%)	19 (5.8%)	4 (3.4%)	0 (0.0%)	2 (1.9%)	0 (0.0%)	**120 (4.9%)**
>20–30	4 (33.3%)	126 (29.0%)	27 (29.7%)	492 (39.9%)	109 (33.5%)	35 (29.9%)	43 (40.6%)	28 (27.2%)	1 (6.7%)	**865 (35.5%)**
>30–40	4 (33.3%)	127 (29.3%)	19 (20.9%)	337 (27.3%)	97 (29.8%)	45 (38.5%)	34 (32.1%)	31 (30.1%)	0 (0.0%)	**694 (28.5%)**
>40–50	0 (0.0%)	66 (15.2%)	20 (22.0%)	198 (16.0%)	58 (17.8%)	23 (19.7%)	18 (17.0%)	25 (24.3%)	1 (6.7%)	**409 (16.8%)**
>50	3 (25.0%)	86 (19.8%)	21 (23.1%)	146 (11.8%)	38 (11.7%)	9 (7.7%)	11 (10.4%)	13 (12.6%)	0 (0.0%)	**327 (13.4%)**
(Missing)	0 (0.0%)	0 (0.0%)	0 (0.0%)	0 (0.0%)	4 (1.2%)	1 (0.9%)	0 (0.0%)	4 (3.9%)	13 (86.7%)	**22 (0.9%)**
NP/OP RT-PCR *										
negative	10 (83.3%)	390 (89.9%)	69 (75.8%)	1037 (84.0%)	288 (88.6%)	100 (85.5%)	100 (94.3%)	31 (30.1%)	7 (46.7%)	**2032 (83.4%)**
positive	2 (16.7%)	37 (8.5%)	7 (7.7%)	110 (8.9%)	35 (10.8%)	17 (14.5%)	6 (5.7%)	10 (9.7%)	5 (33.3%)	**229 (9.4%)**
(Missing)	0 (0.0%)	7 (1.6%)	15 (16.5%)	87 (7.1%)	2 (0.6%)	0 (0.0%)	0 (0.0%)	62 (60.2%)	3 (20.0%)	**176 (7.2%)**
**Serostatus**										
negative	11 (91.7%)	363 (83.6%)	70 (76.9%)	1000 (81.0%)	260 (80.0%)	71 (60.7%)	77 (72.6%)	87 (84.5%)	4 (26.7%)	**1943 (79.7%)**
positive	1 (8.3%)	71 (16.4%)	21 (23.1%)	234 (19.0%)	65 (20.0%)	46 (39.3%)	29 (27.4%)	16 (15.5%)	11 (73.3%)	494 (20.3%)

*Nasal-Oropharyngeal (NP/OP) swab, Reverse Transcription Polymerase Chain Reaction (RT-PCR)

**Figure 7.**
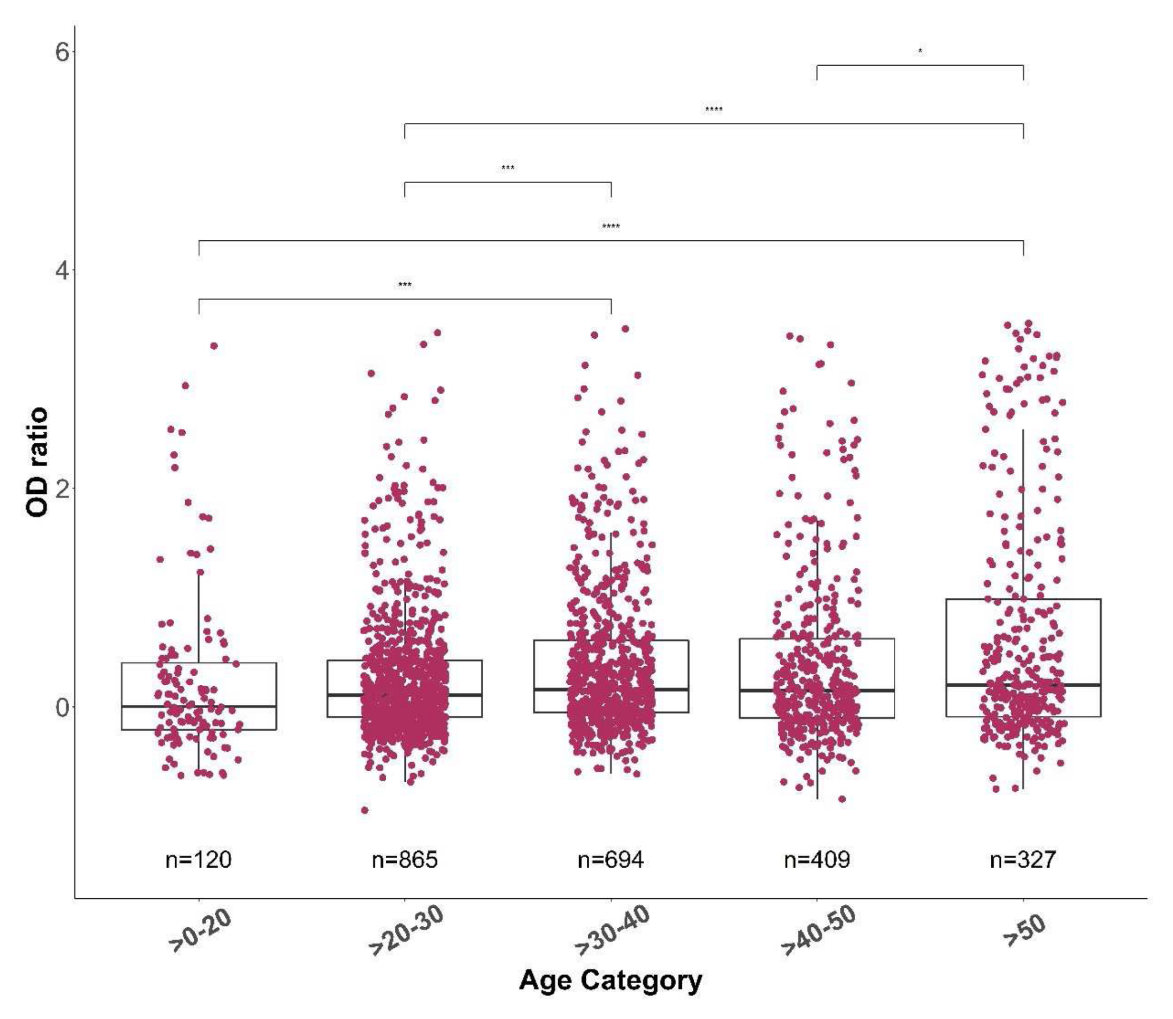
The anti-spike IgG optical density (OD) ratio across different age groups. The median spike OD ratio for each age group was 0.088, 0.156, 0.219, 0.198 and 0.323 for >0-20, >20-30, >30-40, >40-50 and >50, respectively. The total number of samples in each age group is indicated. The OD ratios between each group were statistically compared using a Wilcoxon test. The p-values are shown only for the groups with a significant difference.

**Figure 8.**
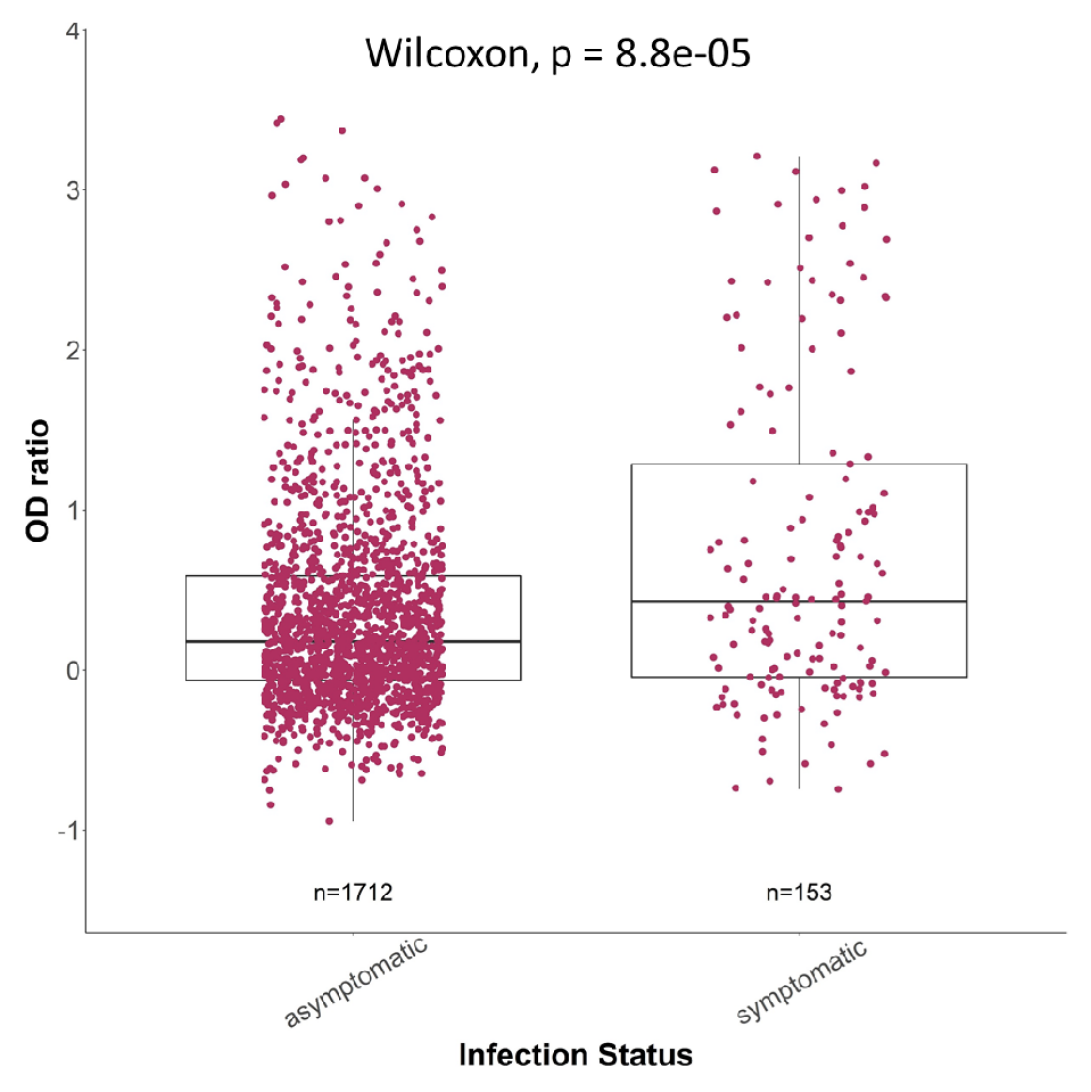
The Spike IgG levels for both asymptomatic (n = 1712) and symptomatic (n = 153) infections. The box plots show the median (middle line) and first and third quartiles (boxes). The P values following a Wilcoxon test are depicted to determine if there is a statistically significant difference in each group.

Individuals in the oldest age group (>50 years) and the youngest age group (>0–20 years) had the highest and the lowest antibody responses respectively ( Figure 7). The antibody responses generally increased with age, with individuals >50 years showing significantly higher antibody responses than all the other age groups ( Figure 7). Furthermore, there were significantly higher antibody responses in symptomatic individuals than asymptomatic individuals (p<0.001, Figure 8). Taking into consideration all the data together, it appears that age and viral load are more likely to result in a change in spike IgG antibody levels ( Table 5).

**Table 5.** A linear regression model to determine whether age, sex, infection status and Ct value, cause a change in OD ratio.

	*Estimate*	*Std. Error*	*t value*	*Pr(>|t|)*	*Significance level*
*(Intercept)*	-0.96398	0.207112	-4.654	6.18E-06	***
*Estimated Age (Years)*	0.007799	0.002905	2.684	0.00792	**
*Sex (Male)*	0.012251	0.088153	0.139	0.88963	
*Infection Status (Symptomatic)*	0.0415	0.106939	0.388	0.69841	
*Ct value (Viral load)*	0.041121	0.006653	6.181	3.98E-09	***

Significance codes: 0 ‘***’ 0.001 ‘**’ 0.01 ‘*’ 0.05 ‘.’ 0.1 ‘ ’ 1. Residual standard error: 0.5728 on 185 degrees of freedom. The Multiple and Adjusted R-squared 0.229 and 0.2124, respectively. F-statistic: 13.74 on 4 and 185 DF, p-value: 7.89e-10. Optical Density (OD), Cycle threshold (Ct)

## Discussion

We report a predominantly asymptomatic case burden (92.2%) along the Coast of Kenya, spread across three waves of peaks in disease transmission. However, since the symptoms were majorly self-reported during mass-testing, or by travellers and truck drivers, the data may be skewed towards the asymptomatic population. The proportion of SARS-CoV-2 asymptomatic infections from other populations around the world range from 5.54% to 75% ( He *et al.,* 2021; Yanes-Lane *et al*., 2020; Zheng *et al*., 2020). It is also possible some of the individuals classified as asymptomatic were pre-symptomatic at the time of reporting and would become symptomatic on follow-up. He *et al*. (2021) have reported 48.9% of pre-symptomatic patients categorized as asymptomatic at the time of screening developing symptoms on follow-up ( He *et al*., 2021). Furthermore, because of our reliance on presentation to routine testing services we cannot be sure why so few individuals present with symptomatic disease. This could reflect reluctance in the population to present for testing when mildly symptomatic or could reflect that symptoms are relatively rare in our population. 

However, we can conclude that asymptomatic infection is very common in our setting, and other lines of evidence confirm widespread transmission in Kenya without overwhelming the health system or high numbers of deaths ( Brand *et al*., 2021).

In contrast to 2020 when the Wuhan strain was predominant, the introduction of new variants in the population such as the Alpha, Beta and Delta variants in 2021 led to an increased proportion in symptomatic individuals but with no clear change in the type of symptoms ( Figure 2 and Figure 3) unlike in the United Kingdom where the Delta variant was associated with predominantly heavy cold and coryza ( Burki, 2021). We did not detect any shift in the type of prevalence of symptoms over time in our population.

The percentage seropositive in the asymptomatic and symptomatic groups in our study was about 45% and 60%, respectively, consistent with proportions reported by Jiang *et al*. (2020), 31–54% to 21–73% seropositivity among asymptomatic and symptomatic individuals respectively ( Jiang *et al*., 2020). However higher seropositivity has been reported by others among RT-PCR positive samples but in all cases, like our case there was more seropositivity in the symptomatic than the asymptomatic groups ( Chen *et al*., 2021; Long *et al*., 2020). We also observed a higher viral load in the symptomatic than the asymptomatic group consistent with other studies ( Chen *et al*., 2021; Jiang *et al*., 2020; Long *et al*., 2020).

Hypotheses to explain the higher proportion of asymptomatic infections in Africa include protective trained immunity and a younger population structure ( Ghosh *et al.,* 2020). Kenya, in common with many other African countries, has a relatively young population compared to high income countries which would directly influence the course of COVID-19 ( Ghosh *et al*., 2020).

There were significantly higher males than females testing positive for COVID-19 which is not unique to this population. Reasons for sex imbalances in COVID-19 datasets include higher expression of angiotensin-converting enzyme-2 ( Jin *et al*., 2020), lifestyle factors such as smoking and alcohol use, and possible variation in adherence to frequent handwashing and wearing of face masks ( Bwire, 2020). Given the predominance of asymptomatic infection in our dataset, a bias towards male involvement with essential services such as truck driving and tendency to travel is likely more relevant ( Kagucia *et al*., 2021).

This study has limitations such as missing data regarding sex, age and clinical features of the tested individuals. Since most of the samples were from surveillance activities, there were not many matched sera/ plasma samples limiting the numbers available for stratified analysis, for example the were few symptomatic individuals and this can skew interpretation of data leading to conclusions which do not reflect the trends in the general population. Data on symptoms were self-reported by patients, hence may be inaccurate due to recall bias.

Nevertheless, our data show clearly the predominance of asymptomatic testing in routine health systems in Kenya, and that asymptomatic infection has been very widespread throughout the course of the epidemic in Kenya.

## Data availability

### Underlying data

The underlying data are owned by the Kenyan Government through the Ministry of Health and as the data contains highly sensitive and confidential information relating to participants, the authors are not permitted to share the data directly. Users who wish to reuse the source data are able to make a request through the KEMRI-Wellcome Trust Research Programme data governance committee: dgc@kemri-wellcome.org. 

### Extended data

Harvard Dataverse: Replication Data for: Epidemiology of COVID-19 infections on routine PCR and serology testing in Coastal Kenya. https://doi.org/10.7910/DVN/BF3BEF ( Nyagwange *et al*., 2022b).

This project contains the following extended data:

- Kenya-COVID19_CIF.pdf (case investigation form)
- Nyagwange_DATASET_Codebook.pdf
- Nyagwange_DATASET_Codebook.xlsx
- Nyagwange_SeroEpidemiology of SARS-CoV-2_Script.Rmd (analysis code)

Data are available under the terms of the Creative Commons Attribution 4.0 International license (CC-BY 4.0).
