# Supplementary material for: Epidemiology of COVID-19 infections on routine polymerase chain reaction (PCR) and serology testing in Coastal Kenya
Source: Wellcome Open Res. 2022 Feb 23;7:69. [Version 1] doi: 10.12688/wellcomeopenres.17661.1 (PMC9034174; doi:10.12688/wellcomeopenres.17661.1)
Supplement: Peer review history

# Peer review: Reviewer response for version 1

**DOI:** 10.21956/wellcomeopenres.19538.r49059

**Published:** 2022-04-22

**Authors:** Maryam Meskini

**Affiliations:** 1 Microbiology Research Center, Pasteur Institute of Iran, Tehran, Iran; Mycobacteriology & Pulmonary Research Department, Pasteur Institute of Iran, Tehran, Iran

The study was well done and well written. I think it would be better to  consider some criteria such as vaccination index and the number of doses injected which could change the conclusion.

 Please check the following comments to improve the level of your manuscript:

1. Please write the title in capital letters.
2. Please don’t use an abbreviation for the first time and write in full. E.g., SARS at the second line of the abstract.
3. Please provide the high-resolution format of tables and figures.
4. Many of the sentences don’t have a reference(s). Please mention the reference at the end of each sentence or paragraph.
5. Some sentences are repetitive. Please don’t repeat a concept in different sentences.
6. Please check the grammatical of the manuscript.
7. I think it is better to consider criteria such as vaccination index and the number of injected doses in the subjects in the study, which will probably change the results and consequently change the conclusion. In other words, it is better to consider that some people who were asymptomatic but were found to be positive for COVID-19 by molecular technique (RT-PCR) were vaccinated and injected with several doses. For instance, can vaccination be given as one of the reasons for asymptomatic patients?

Is the work clearly and accurately presented and does it cite the current literature?

Yes

If applicable, is the statistical analysis and its interpretation appropriate?

I cannot comment. A qualified statistician is required.

Are all the source data underlying the results available to ensure full reproducibility?

Yes

Is the study design appropriate and is the work technically sound?

Yes

Are the conclusions drawn adequately supported by the results?

Yes

Are sufficient details of methods and analysis provided to allow replication by others?

Yes

Reviewer Expertise:

Microbiology, Molecular Biology, Bacteriology

I confirm that I have read this submission and believe that I have an appropriate level of expertise to confirm that it is of an acceptable scientific standard.

# Peer review: Reviewer response for version 1

**DOI:** 10.21956/wellcomeopenres.19538.r49085

**Published:** 2022-03-28

**Authors:** Olusola Anuoluwapo Akanbi

**Affiliations:** 1 Nigeria Centre for Disease Control, Abuja, Nigeria

Authors have conducted a COVID-19 retrospective analysis over the period March 2020 to June 2021 in the Coastal regions of Kenya. The study design as well as the methods for analysis are adequate however, it would be great if authors could get information on vaccination status of participants where available especially for those serum samples collected and also participants reqruited in the year 2021 when COVID-19 vaccination started in Kenya. This I believe will add more value to this work.

Is the work clearly and accurately presented and does it cite the current literature?

Yes

If applicable, is the statistical analysis and its interpretation appropriate?

I cannot comment. A qualified statistician is required.

Are all the source data underlying the results available to ensure full reproducibility?

Yes

Is the study design appropriate and is the work technically sound?

Yes

Are the conclusions drawn adequately supported by the results?

Yes

Are sufficient details of methods and analysis provided to allow replication by others?

Yes

Reviewer Expertise:

Microbiology, Virology, Genomics, Molecular Biology

I confirm that I have read this submission and believe that I have an appropriate level of expertise to confirm that it is of an acceptable scientific standard.
